# Identifying the barriers and enablers for a triage, treatment, and transfer clinical intervention to manage acute stroke patients in the emergency department: a systematic review using the theoretical domains framework (TDF)

**DOI:** 10.1186/s13012-016-0524-1

**Published:** 2016-11-28

**Authors:** Louise E. Craig, Elizabeth McInnes, Natalie Taylor, Rohan Grimley, Dominique A. Cadilhac, Julie Considine, Sandy Middleton

**Affiliations:** 1 Nursing Research Institute, St Vincent’s Health Australia (Sydney) and Australian Catholic University, Sydney, NSW Australia; 2 Centre for Healthcare Resilience and Implementation Science, Australian Institute of Health Innovation, Macquarie University, Sydney, NSW Australia; 3 Sunshine Coast Hospital and Health Service/Sunshine Coast Clinical School, The University of Queensland, Nambour, QLD Australia; 4 Translational Public Health and Evaluation Division, Stroke and Ageing Research, School of Clinical Sciences at Monash Health, Monash University, Clayton, VIC Australia; 5 Public Health: Stroke Division, Florey Institute of Neuroscience and Mental Health, University of Melbourne, Parkville, VIC Australia; 6 Deakin University, Geelong, VIC Australia; 7 Eastern Health – Deakin University Nursing and Midwifery Research Centre, Box Hill, VIC Australia

**Keywords:** Implementation, Barriers, Enablers, Theoretical domains framework, Acute stroke, Emergency department

## Abstract

**Background:**

Clinical guidelines recommend that assessment and management of patients with stroke commences early including in emergency departments (ED). To inform the development of an implementation intervention targeted in ED, we conducted a systematic review of qualitative and quantitative studies to identify relevant barriers and enablers to six key clinical behaviours in acute stroke care: appropriate triage, thrombolysis administration, monitoring and management of temperature, blood glucose levels, and of swallowing difficulties and transfer of stroke patients in ED.

**Methods:**

Studies of any design, conducted in ED, where barriers or enablers based on primary data were identified for one or more of these six clinical behaviours. Major biomedical databases (CINAHL, OVID SP EMBASE, OVID SP MEDLINE) were searched using comprehensive search strategies. The barriers and enablers were categorised using the theoretical domains framework (TDF). The behaviour change technique (BCT) that best aligned to the strategy each enabler represented was selected for each of the reported enablers using a standard taxonomy.

**Results:**

Five qualitative studies and four surveys out of the 44 studies identified met the selection criteria. The majority of barriers reported corresponded with the TDF domains of “environmental, context and resources” (such as stressful working conditions or lack of resources) and “knowledge” (such as lack of guideline awareness or familiarity). The majority of enablers corresponded with the domains of “knowledge” (such as education for physicians on the calculated risk of haemorrhage following intravenous thrombolysis [tPA]) and “skills” (such as providing opportunity to treat stroke cases of varying complexity). The total number of BCTs assigned was 18. The BCTs most frequently assigned to the reported enablers were “focus on past success” and “information about health consequences.”

**Conclusions:**

Barriers and enablers for the delivery of key evidence-based protocols in an emergency setting have been identified and interpreted within a relevant theoretical framework. This new knowledge has since been used to select specific BCTs to implement evidence-based care in an ED setting. It is recommended that findings from similar future reviews adopt a similar theoretical approach. In particular, the use of existing matrices to assist the selection of relevant BCTs.

## Background

Clinical guidelines recommend that the assessment and management of patients with stroke should commence early in the pre-hospital setting and hospital emergency department (ED) [1]. Despite the recent advances in interventions for the management of acute stroke, only a small proportion of individuals receive recommended evidence-based treatment in the hours following acute stroke [1, 2]. For example, thrombolysis using intravenous tissue plasminogen activator (tPA) is currently one of the few evidence-based treatments available for acute ischaemic stroke, however, internationally rates are variable, ranging from 5% in the USA [3] to 14% in some European Centres [4]. Furthermore, outcomes are improved by early administration (within hours) of tPA after symptom onset. However, in-hospital delays are often a significant obstacle in achieving early administration of tPA [5].

Determining the inhibiting factors (barriers) and supporting factors (enablers) for implementation of research evidence, is a well-established requirement to improve the quality of patient care [6]. It has been demonstrated that a theoretical approach to assessing barriers and enablers can effectively be used for developing tailored informed strategies to support the effective implementation of evidence-based practices, such as hand hygiene [7]. Systematic reviews offer a way to synthesise the broad range of barriers and enablers reported in individual studies and provide a broader understanding of the influences on evidence-based treatment uptake. Findings from such reviews can be used to inform the development of effective interventions to implement evidence-based care in clinical settings.

In stroke, systematic reviews have been conducted to identify barriers and enablers to implementing elements of stroke guidelines. These have usually been studied at an organisational level and have included pre-admission barriers such as non-recognition of stroke [8], triaging of stroke as non-urgent by both ED and ambulance staff [8, 9], delays in accessing imaging [8] and inefficient hospital processes and protocols [8, 9]. Data included in these reviews were based on retrospective analyses of hospital databases [5, 10] national registries [11], or prospective cohort data. The main limitation of these reviews is that the barriers and/or enablers were based on the authors’ perceptions or explanations rather than based on healthcare staffs’ perceptions or beliefs.

Recently, within the implementation science literature there has been increasing importance placed on the development of behaviour change interventions [12, 13] using theoretical models or frameworks such as the Theoretical Domains Framework (TDF) [14]. The use of theory is important to understand the factors that influence healthcare professionals’ behaviours, to inform the use of possible behaviour change techniques (BCTs) and to provide clarity as to how these techniques might work [15, 16]. Interventions are said to be more effective if interventions are based on evidence-based principles drawn from theories of behaviour and behaviour change [17].

The TDF is a framework of originally 12 domains [14] (now 14 domains [18]). The theoretical domains were derived from 33 behaviour change theories and developed using a process of expert consensus with subsequent validation work. To facilitate the application of BCT taxonomy and to assist the selection of relevant BCTs a matrix has been developed which is based on identifying links between specific BCTs and theoretical constructs such as those used in the TDF [19]. The TDF has been used in a number of healthcare settings to study implementation and more specifically assist in the development of implementation interventions [16]. The TDF has also been used as a coding framework for the analysis of barriers in systematic reviews [20–22]. In one study the TDF allowed the researchers to explore, explain and potentially target, using sophisticated behaviour change interventions, complex relationships, for example, limited “knowledge” appeared to influence healthcare professional’s ‘emotions’ [21].

In the Quality in Acute Stroke Care Study (QASC) the investigators demonstrated significant benefits for patients who were cared for in acute stroke units (ASU) in which staff had received support for implementation of protocols to manage fever, hyperglycaemia, and swallowing dysfunction [23]. The QASC trialists recommended future trials to examine the multidisciplinary intervention in other settings, such as the ED, to ensure patients had rapid access to these evidence-based protocols [23]. Prior to the development of an implementation intervention to deliver such evidence-based protocols, we undertook a systematic review of qualitative and quantitative studies to identify the barriers and enablers specific to an ED setting. The findings from this systematic review will inform the use of specific BCTs to develop an implementation intervention.

### Aim

The aims of this systematic review were to:

- Identify the reported barriers and enablers to implementing the following evidence-based care elements (hereonin referred to as target clinical behaviours): appropriate triage, thrombolysis administration, monitoring and management of temperature, blood glucose levels, and of swallowing difficulties and rapid (within 4 h of arrival to ED) transfer to the ASU
- Classify reported barriers and facilitators using the TDF
- Select the BCT that best aligned to the strategy each enabler represented using standard taxonomy

## Methods

### Inclusion and exclusion criteria

Studies were eligible for inclusion, regardless of design, if the study:

- Aim was to identify the barriers and/or enablers for any one or a combination of the target clinical behaviours in the ED of a hospital (Table 1) and
- Included the views/perceptions of healthcare professionals regarding the target behaviours who worked in ED.

**Table 1:** Target clinical behaviours

Clinical behaviour	Description
Triage	All patients presenting with signs and symptoms of suspected acute stroke should be triaged as Australian Triage Scale Category or 2 (seen within 10 mins)
Thrombolysis	All patients to be assessed for tPA eligibility All eligible patients to receive tPA
Management of temperature	All patients to have their temperature taken on arrival to Emergency Department (ED) and then at least four hourly whilst they remain in ED Temperature 37.5 °C or greater to be treated with paracetamol (acetaminophen) within one hour
Management of blood glucose levels	Venous blood glucose level (BGL) sample sent to laboratory on admission to ED Finger prick BGL recorded on admission and finger prick BGL monitored every 6 h (or greater if elevated) Insulin administered to all patients with BGL > 10 mMol/L within one hour
Swallow assessment	Patients to remain nil by mouth until a swallow screen by non- Speech Pathologist (SP) or swallow assessment by SP performed All patients who fail the screen to have a swallowing assessment by a SP
Transfer	All patients with stroke to be discharged from ED within 4 h All patients with stroke to be admitted to the hospital’s stroke unit

Abstracts, letters, editorials, and commentaries were excluded. No restrictions were placed on country, written language, or year of publication.

### Search methods for identification of studies

The search strategy was developed using search concepts (groups of words; Appendix). Potentially relevant studies were identified through a search (inception to August 2016) of the following electronic databases: CINAHL, OVID SP EMBASE, OVID SP MEDLINE, and Web of Science. Other databases that were searched included: OVID SP PubMed Central; The Joanna Briggs Institute EBP; Database ProQuest Dissertations & Theses Full Text. The following grey literature databases also were searched: Agency for Healthcare Research and Quality (AHRQ); Open Grey and Grey Literature Report.

The Science Citation Index (Web of Science) was searched to identify further studies that had cited the studies included in the review. Reference lists of included publications were searched to identify additional studies.

### Search strategy

The search strategy applied to the databases was a combination of Medical Subject Headings (MeSH) terms such as: “Health Plan Implementation” and “Evidence-Based Practice”, as well as additional keywords such as “barrier”, “uptake,” and “enabler”, and relevant synonyms. The MeSH terms and keywords were generated for each of the search concepts by examining the terminology and database indexing used in relevant papers. The search strategies were reviewed by a University Librarian with experience in database searches, prior to the search being undertaken.

### Screening process

The titles and abstracts of retrieved references from the search were screened by a single reviewer to exclude obviously irrelevant studies. The full article of any study that met the inclusion criteria was reviewed by at least two of the authors (EM, LC, or SM).

### Data extraction

Data were extracted using a standardised form by one reviewer (LC), with a sub-set of included papers (*n* = 15 [30%]) being extracted by a second reviewer (EM or SM). Data collection included full study characteristics such as author, date of publication, study design and the reported study findings, i.e., barriers and/or enablers. Barriers and/or enablers based on primary data were extracted using theme headings and theme descriptions in qualitative studies and extracted from tables presenting questionnaire responses to pre-specified barriers and/or enablers in the quantitative studies (Table 2). Six of the nine included studies [24–29] focused on one target behaviour which was easily identifiable from the title on screening and the aim and method sections on data extraction. The remaining three studies [30–32] focused on stroke pathways or key stroke care recommendations whereby the target behaviours of interest were identified in the methods and results sections of the individual studies. If the lead author was unclear which target behaviour the data represented this was cross-checked and discussed with another author (SM or EM). Authors of included studies did not distinguish between modifiable and non-modifiable barriers/enablers.

**Table 2:** Characteristics of included studies

Author/date	Aim of study	Design	Method of data collection	Source of barrier/enabler data extraction	Participants
Daniels et al. (2013) [24] USA	To identify strategies for effective implementation of swallowing screening in patients with stroke symptoms that presented in ED	Qualitative	Staff interviews	Barrier and enabler themes	ED nurses (*n* = 8)
Gache et al. (2014) [30] France	To identify the main barriers to effective implementation of Stoke Care Pathway in France	Qualitative	Semi-structured interviews	Barrier typology derived from data	Emergency physicians, neurologists, geriatricians, social workers, health care workers in rehab and nursing homes (*n* = 33)
Grady et al. (2014) [25] Australia	To assess emergency physicians’ perceptions of individual and system enablers to the use of thrombolysis in acute stroke	A web-based survey	Questionnaire	Responder’s agreement to pre-defined enabler statements	Australian fellows and trainees registered with ACEM (*n* = 429)
Hargis et al. (2015) [26] USA	To identify factors that may limit the administration of rt-PA in the emergency department at multiple stroke centres	A web-based survey	Questionnaire	Responder’s agreement to pre-defined enabler statements	ED nurses and pharmacists (*n* = 37)
Johnson MJ et al. (2011) [31] USA	To describe emergency nurses’ perceptions of specific barriers and enablers to the care of stroke patients in the emergency department	Qualitative	Focus groups	Barrier and enabler themes	Emergency nurses currently employed in an emergency department (*n* = 10)
Meuer et al. (2011) USA	To describe the pre-identified barriers to clinicians compliant with guidelines recommending the use of thrombolysis	Qualitative	Focus groups and one-to-one interviews	Barrier listed in the coding guide with definitions	Emergency physicians, nurses, neurologists, radiologists, hospital administrators, and hospitalists and pharmacist (*n* = 30)
Skecksen A et al. (2014) Sweden	To identify and analyse the barriers and enablers to implementing national thrombolytic guidelines	Qualitative	Semi-structured interviews	Barrier and enabler themes	Stroke healthcare professionals (nurses and physicians) (*n* = 16)
Van Der Weijden et al. (2004) [32] The Netherlands	To explore the opinion on possible barriers for working according to key recommendations for the acute phase a stroke care among neurologists	Paper-based survey	Questionnaire	Responder’s agreement to pre-defined barrier statements	Registered neurologists (*n* = 16)
Williams J et al. (2013) [29] Australia	To identify barriers which prevent rural health care providers from utilising thrombolysis in acute ischamic stroke	Paper-based survey	Questionnaire	Responder’s agreement to pre-defined barrier statements	All rural sites within NSW Australia that had an implemented thrombolysis service as defined by the NSF and an Stroke Care Coordinator position were deemed eligible for inclusion (*n* = 11)

*ACEM* Australasian College for Emergency Medicine, *ED* Emergency Department, *NSF* National Stroke Foundation

#### Qualitative studies

Verbatim supporting quotes, where available, were extracted to illustrate the barriers and enablers. Author interpretations or findings from secondary analysis of routine data were not included.

#### Quantitative studies

To ensure that the review presented findings that were representative and important, certain decisions were made about the data extraction for the survey data. Pre-specified barriers where zero or only one of the participants selected were not extracted [26, 29]. A decision was made to extract only the pre-specified barriers where the majority of participants agreed (>50%) for one study [25] and a further decision was made to extract pre-specified barriers which scored a level of agreement >3 (1 = fully disagree; 5 = fully agree) for the remaining survey [32].

### Quality assessment

Quality assessment of qualitative studies was conducted using the Critical Appraisal Skills Programme Qualitative Checklist (CASP) [33]. For the assessment of quantitative studies the Centre for Evidence-Based Management “Appraisal of a Survey” tool was used [34]. Critical appraisal was conducted by one reviewer (LC) for all studies, with second reviewer appraisal (EM or SM) for a sub-set of included papers (the same sub-set subject to second reviewer data extraction). The findings from the two reviewers were compared and any contrasting items were discussed and re-reviewed to reach an agreement.

### Data analysis

The extracted data were classified using the TDF [18]. Classification of barriers and enabler data was conducted independently by two researchers experienced in the application of the TDF (LC and NT). Reference was made to the original article regarding the development of the TDF to ensure accurate interpretation of the domains [14]. The TDF constructs [18] and contextual information reported for an individual barrier/enabler were also used to allocate the data to the most appropriate domain. Using the descriptors of each TDF domain, the individual barriers and enablers were classified accordingly. There were two disagreements between the two researchers resulting in the re-classification of two barriers, one from the *beliefs about capabilities* domain to the *social influences* domain and the other from the *beliefs about capabilities* domain to the *intentions* domain. The TDF was used as a relevant framework to narratively summarise the individual barriers under the relevant theoretical domains; but no thematic synthesis was conducted to identify themes.

#### Allocating BCT labels to reported enablers

A matrix which assigns the most appropriate BCT to each of the TDF domains has already been developed by Cane et al. [35]. Primarily, this resource has been used in the past to develop behaviour change interventions to address identified key barriers. However, the studies included in our review did not report strategies to overcome specific barriers, so BCTs could not be assigned. However, the enablers reported in each study were able to be aligned with BCTs listed in the Cane matrix, as each reported enabler essentially represented a strategy to promote behaviour. The BCT that best aligned to the enabling strategy was then selected for each of the reported enablers using this matrix [36]. The assignment of BCTs to enablers was independently conducted by two researchers. Both researchers then discussed the allocations collectively and resolved any disagreements by discussion. This approach to classifying enablers to BCT enhances reporting as it provides a standardised label for the reported enabler using a common BCT taxonomy and subsequently increases the transferability of the findings.

## Results

**Fig. 1:**
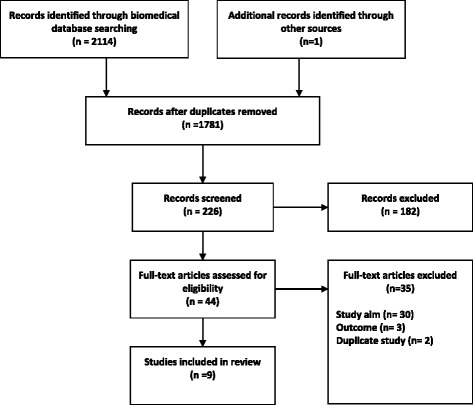
Search flow

The search identified 2114 studies. Following the initial screening of titles and abstracts, duplicate and irrelevant studies were excluded, and the full-text articles of 44 studies were assessed in detail (Fig. 1). One further article was identified through citation searching. No further articles were identified from reviewing the reference lists of the included studies. Overall, nine studies met the selection criteria and were included in the review [24–32].

### Study characteristics

Table 2 shows the characteristics of the nine studies which were all published between 2004 and 2015. Five studies were qualitative with one using focus group methods [31] and four using semi-structured interviews [24, 27, 28, 30]. The remaining four studies used a survey method, of which two used an online questionnaire [25, 26]. The sample sizes ranged from 8 to 429 healthcare professionals. Four studies were conducted in the USA, two in Australia, one in Sweden, one in the Netherlands, and one in France. Sampling strategies to recruit participants varied. In five studies a convenience sample was used [24, 25, 29, 31, 32] and in four studies a purposive sampling strategy was used [26–28, 30].

In one study, barriers and enablers relating to the triage behaviour were identified [31]. In four studies, barriers and enablers relating to the tPA related behaviours were reported [25, 27–29]. One study only reported barriers (i.e. no enablers) to the tPA related behaviours [26] and one study reported the barriers for both the tPA and transfer related behaviours [32]. In another study, the barriers and enablers relating to both the tPA and the triage related behaviours were reported [30]. Lastly, barriers and enablers to the swallow assessment behavior were reported in a single study [24]. None of the included studies provided evidence on the barriers and enablers for behaviours relating to management of fever, or for the management of blood glucose levels.

### Quality of the included studies

The common limitations within the qualitative studies were: no or little information about the relationship between the researcher and participants [24, 27, 28, 30, 31], and a lack of data describing and justifying the approach for analysis [24, 28, 31]. Generally, the strengths of the studies were clearly stated aims, appropriate use of methods and a clear statement of findings (Table 3).

**Table 3:** Quality assessment results of qualitative included studies

Quality assessment question	Daniels et al., 2013 [24]	Gache et al., 2014 [30]	Johnson et al., 2011 [31]	Meuer et al., 2011	Skecksen et al., 2014
Was there a clear statement of the aims of the research?	✓	✓	✓	✓	✓
Is a qualitative methodology appropriate?	✓	✓	✓	✓	✓
Was the research design appropriate to address the aims of the research?	✓	✓	✓	✓	✓
Was the recruitment strategy appropriate to the aims of the research?	✓	✓	✓	✓	x
Was the data collected in a way that addressed the research issue?	✓	✓	✓	✓	✓
Has the relationship between researcher and participants been considered?	x	x	Not reported	x	x
Have ethical issues been taken into consideration?	✓	✓	Not reported	✓	✓
Was the data analysis sufficiently rigorous?	x	✓	Not reported	✓	x
Is there a clear statement of findings?	✓	✓	Not reported	✓	✓
How valuable is the research?	✓	✓	✓	✓	✓

X = No; ✓ = Yes

With regard to the quality of the quantitative research (Table 4), the low response rates (13–55%) limits the potential generalisability of these studies. All of these studies had a clearly defined research question.

**Table 4:** Quality assessment results of quantitative included studies

Quality assessment question	Grady et al., 2014 [25]	Hargis et al., 2015 [26]	Williams J et al., 2013 [29]	Van Der Weijden et al., 2004 [32]
Did the study address a clearly focused question/issue?	✓	✓	✓	✓
Is the research method appropriate?	✓	x	x	x
Is the method of selection of the subjects clearly described?	✓	✓	✓	✓
Could the way the sample was obtained introduce bias?	Not reported	x	Not reported	x
Was the sample of subjects representative with regard to the population?	✓	✓	Not reported	✓
Was the sample size based on considerations of statistical power?	x	x	x	✓
Was a satisfactory response rate achieved?	x	✓	x	✓
Are the measurements likely to be valid and reliable?	Not reported	✓	Not reported	✓
Was the statistical significance assessed?	✓	x	x	x
Are confidence intervals given for the main results?	x	x	x	✓
Could there be confounding factors that haven’t been accounted for?	x	x	x	Not reported
Can the results be applied to your organisation?	Not reported	✓	Not reported	✓

X = No; ✓ = Yes

### Participant characteristics

The nine included studies provided data from 590 healthcare professionals working in a hospital ED. In two studies, the mean age of participants was reported (41.1 years [25] and 33.9 years [31]). Out of the four studies where the sex of participants was provided, the percentages that were female ranged from 13.5% [32] to 100% [31]. The type of participants were all nurses in two studies [24, 31], physicians and nurses in the remaining seven studies, of which four of these included participation by other disciplines such as social workers or pharmacists [25–27, 30]. The length of experience working in the ED was reported in two studies and ranged from the majority of participants having worked in ED over 10 years [25] and between 1 month and less than 17 months [30].

### Domains of the TDF as represented by the reported barriers and enablers

A summary of barriers and enablers by TDF domain as identified in each of the included studies is provided in Table 5. The review identified 51 barriers and 40 enablers relevant to the target clinical behaviours. The number of barriers and number of enablers reported for triage behaviour were nine and one, respectively. The number of barriers and number of enablers reported for the thrombolysis related behaviours were 21 and 36, respectively. The number of barriers and number of enablers reported for swallow assessment behaviour were four and three, respectively. Only one barrier was reported for transfer-related behaviours.

**Table 5:** Table of findings by TDF domain

Target clinical behaviour	TDF domain	Reported barrier	Reported enabler	Behaviour change technique label
Swallow assessment	Environmental context and resources	- Difficulty finding time to document screening results in the electronic health record [24]	- Efficient processes to support swallow screen tool administration and interpretation [24]	- Restructuring the physical environment
	Social influences	- No data available	- Multidisciplinary team cooperation and support from ED administrators [24]	- Social support (unspecified)
	Knowledge	- No data available	- More education on dysphagia and evidence-based screening of swallowing [24]	- Information about health consequences
	Skills	- Inaccurate interpretation of screening items [24] - Inconsistent administration of the swallow screen tool [24]	- No data available	- No data available
	Memory, attention and decision processes	- Difficulty recalling all screening items during administration of the swallow screen tool [24]	- No data available	- No data available
All patients to be assessed for tPA eligibility All eligible patients receive tPA	Beliefs about capabilities	- Lack of self-efficacy [27]	- Informants emphasized that the rapid expansion of stroke treatment options in recent decades has contributed to work pride and improved motivation to implement guidelines [28] - “I can” accurately identify stroke patients (93.7%) [25] - “I can” accurately identify which stroke patients may be eligible for tPA (62.0%) [25] - The hospital has a policy for the management of stroke patients (85.8%) [25] - The hospital has a policy for rapid referral of suspected stroke patients from ED to stroke specialists (76.2%) [25] - The hospital has a policy for rapid access to imaging for suspected stroke patients (87.0%) [25] - The hospital has a policy for administration of tPA when appropriate (72.7%) [25] - Exposure, mentoring, protocols and experience through the implementation of stroke units in rural facilities, telemedicine and stroke code protocols might be beneficial to improve physicians’ ability to confidently diagnose stroke patients eligible for tPA treatment [29] - Confidently interpret brain imaging scans (66.9%) [25]	- Social support (practical)^a^ - Focus on past success - Focus on past success - Focus on past success - Focus on past success - Focus on past success - Focus on past success - Verbal persuasion about capability; Focus on past success; Exposure^a^ - Focus on past success
	Intentions	- Lack of motivation [27]	- Taking active part in quality improvement and research programs [28]	- Restructuring the social environment^a^
	Knowledge	- Lack of guideline awareness [27] - Lack of guideline familiarity [27] - Lack of knowledge about and experience with thrombolytic therapy [28] - Failure to react to guideline deviations [28] - Uncertainty with patient selection criteria [29] - Blood pressure control [26]	- Guideline awareness and knowledge among all staff [28] - Knowledge and attitudes of the providers on how to offer tPA to stroke patients [27] - Continuing professional education [28] - Education on symptoms of stroke, tPA use, pathways and protocols, its efficacy and ICH risk [29] - Education for physicians on the calculated risk of ICH following intravenous tPA [29]	- Information about health consequences - Information about health consequences - Information about health consequences - Information about health consequences - Information about health consequences
	Environmental context and resources	- Lack of agreement between guidelines [27] - Stressful working conditions [28] - Recruitment difficulties [28] - Limited time, human, and financial resources [28] - Duty schedule inhibiting training [28] - Lack of continuity (with various dimensions) [28] - Pre-hospital delays [29] - Patients presenting outside the time window [26] - ED delays [29] - Long communication time between ED staff and neurology team [26] - Delayed referral from GP [32] - Not ideal setting [29] - Administrative barriers [29] - Lack of urgency in the ED [26]	- Formal and informal meetings [28] - Short intra hospital distances for thrombolytic processes [28] - “To help me follow stroke care protocol” there are checklists/decision aids to help identify and triage a possible stroke case (68.8%) [25] - “To help me follow stroke care protocol” there are checklists/decision aids to help identify stroke patients eligible for tPA (66.7%) [25] - “At all times I have immediate access to” advice from a senior colleague in managing stroke (76.9%) [25] - “I have immediate access to” staff trained to interpret images (78.5%) [25]	- Restructuring the social environment - Restructuring the physical environment - Prompts/cues - Prompts/cues - Restructuring the physical environment - Restructuring the physical environment
	Beliefs about consequences	- Lack of outcome expectancy [27] - Old-fashioned views on stroke, with low expectations of therapeutic options [28] - Physician reluctance [26] - Undue respect for treatment [28] - Risk of intra-cranial haemorrhage [29] - Uncertainty about benefits of tPA [29]	- No data available	- No data available
	Social/professional role and identity	- Insufficient recognition by peers and decision makers [28] - Poor professional identity [28] - Formal power structures and prestige [28]	- Close collaboration with staff outside the stroke unit [28] - Good leadership [28]	- Restructuring the physical environment^a^ - Social support (unspecified)
	Optimism		- Positive staff attitudes, within and outside the stroke unit [28]	- No corresponding technique^b^
	Behavioural regulation	- Failure to react to guideline deviations [28]	- Implementation work included in routines [28] - Feedback on success or failure [28] - Quality assurance with continuous feedback on implementation progress [28]	- Habit formation^a^ - Feedback on outcome(s) of behaviour^a^; Feedback on behaviour^a^ - Feedback on outcome(s) of behaviour^a^; Feedback on behaviour^a^
	Skills	- Interpretation of CT [29] - Clinical diagnostic uncertainty [29] - Personal stroke neurology experience [29] - Experience with tPA inclusions and exclusions [29] - Difficulty identifying stroke in presenting patients [26]	- Exposure and experience through the implementation of stroke units in rural facilities, telemedicine and stroke code protocols might be beneficial to improve physicians’ ability to confidently diagnose stroke patients eligible for tPA treatment [29] - Continuing professional education [28] - “I regularly” treat acute stroke patients (91.4%) [25] - “I regularly” have the opportunity to treat stroke cases of varying complexity (88.3%) [25] - Trained stroke nurses available [25] - “I have seen” tPA administered to stroke patients on several occasions (78.8%) [25]	- Behavioural practice/rehearsal - Instruction on how to perform a behaviour^a^ - Behavioural practice/rehearsal - Behavioural practice/rehearsal - Restructuring the physical environment^a^ - Demonstration of the behavior^a^
	Social influences	- Lack of support [28, 29]	- Involvement of all professionals in implementation work [28] - Respected and influential members of this hospital endorse the use of tPA (67.5%) [25] - Between-hospital benchmarking and sharing experiences with staff at other hospitals [28]	- Social support (unspecified) - Information about others’ approval - Social comparison
Triaged at Australian Triage Scale 1 or 2	Knowledge	- Inadequate public education about stroke: including patients and GPs [30] - Stroke not recognised as a priority [31]	- No data available	- No data available
	Environmental context and resources	- Lack of resource : staff shortages in facilities [30] - Competing demands in ED and staffing challenges during busy times [31]	- Having the stroke protocol for consistency [31]	- Prompts/cues
	Skills	- Lack of training and public information [30]	- No data available	- No data available
	Social/professional role and identity	- Lack of coordination between staff [30] - Overlong waiting times – stroke care, examinations [30]	- No data available	- No data available
	Beliefs about capabilities	- Lack of comfort with assessing stroke patients using the National Institutes of Health Stroke Scale [31]	- No data available	- No data available
Transfer	Environmental context and resources	- Poor patient flow to the rehabilitation centre [32]	- No data available	- No data available

*CT* Computed tomography, *ED* Emergency departments, *ICH* Intracerebral Haemorrhage

^a^This technique was not suggested by the Cane et al. matrix for the corresponding domain

^b^It was agreed that there was no behaviour change technique that represented this enabler. This is possibly due to the limited reporting of how the staff were influenced to develop the positive attitudes

#### Knowledge

Barrier and enabler data were available relating to triage and tPA-related behaviours. Both studies investigating triage reported “stroke was not being recognised as a priority” as a barrier [30, 31]. All three studies for the tPA-related behaviours identified barriers associated with lack of knowledge including guideline unfamiliarity [27], awareness [27], or failure to react to deviations in guidelines [28]. The barriers identified by Williams et al. related more to procedural knowledge such as uncertainty with respect to the patient selection criteria for tPA [29]. More specifically, in one study staff (ED nurses and pharmacists) reported that physicians wait until towards the end of the tPA treatment time window to see whether patients’ symptoms improve before committing to tPA, thus delaying time to tPA treatment and potentially lowering tPA treatment rates [26]. Education to provide knowledge about how to offer tPA [27], identification of stroke [25] and interpretation of brain imaging scans was reported as an enabler [25]. Correspondingly, lack of education on symptoms of stroke, tPA use, and lack of pathways and protocols were reported as significant barriers by Williams et al. [29]. More education around dysphagia and evidence-based screening of swallowing post stroke was identified as an enabler by the only study investigating the swallow care behaviours [24].

#### Skills

Lack of skills was reported in relation to the triage, tPA and swallow care behaviours, but not for the transfer behaviours. Staff reported that a lack of practice and skills development were barriers to administrating tPA, particularly with regards to interpretation of computed tomography scans, clinical diagnostic uncertainty and individual experience in stroke neurology [29]. In one study, the administration and inaccurate interpretation of swallow screening items were reported [24].

#### Social/professional role and Identity

Social/professional role and identity related issues were reported for the triage and the tPA-related behaviours. A lack of a dedicated nurse to manage stroke patients was reported in one study [26]. Limited social identity, i.e., insufficient recognition by peers and decision makers were barriers to delivering the tPA-related behaviours [28].

#### Beliefs about capabilities

Data relating to beliefs about capabilities were only reported for the tPA-related behaviours. Barriers relating to this domain included a lack of self-efficacy and motivation [27]. A number of similar enablers, were found in two studies and included experience and exposure of “telemedicine and stroke code protocols”, which were focused on improving physician’s ability to confidently diagnose stroke patients eligible for tPA treatment [29]. Similarly, Grady et al. reported that the majority of survey participants believed that being able to “confidently interpret brain imaging scans” was an enabling factor [25].

#### Optimism

Data were only reported for the tPA-related behaviours and relevant enablers reported by staff included positive staff attitudes within and outside the stroke unit [28].

#### Beliefs about consequences

Data were only reported for tPA-related behaviours. In three studies staff beliefs indicated belief in a lack of positive outcome for patients after tPA [27–29] including that staff held “old-fashioned views” about the lack of benefit of tPA for improving outcomes after stroke [28]. Reluctance of staff to administer tPA was reported (participants were ED nurses and ED pharmacists) in one study as a barrier and this was likely to be due to poorly understood beliefs about the benefits of using tPA [26].

#### Intentions

Data were only reported for the tPA related care elements. Lack of motivation was the only barrier reported which related to this TDF domain [27].

#### Memory, attention, and decision process

Data were only reported for the swallow assessment behaviours. The only factor relating to this domain was staff reporting difficulty recalling all screening items during administration of a swallow screen [24].

#### Environmental context and resources

All care elements apart from the management of blood glucose levels and the management of fever had information reported on environmental context and resources. Working with a busy environment with competing demands was a frequently reported barrier for the appropriate triage of patients with stroke. Barriers were largely related to delays and were often categorised as pre-hospital clinical care, in-hospital clinical care and/or administrative to the successful assessment for and delivery of tPA [26, 29]. Communication between departments was believed to play a pivotal role in the success of delivering tPA [26]. Financial resources were reported as a potential barrier in one study, without further specification [31], but in another, no respondents rated cost as a significant barrier [28]. Stecksen et al. identified stressful working conditions, limited time, and lack of continuity as other barriers to assessing patients for suitability for tPA and delivering tPA treatment [28]. In relation to rapid transfer of stroke patients from the ED to the stroke unit, the two barriers reported related to poor patient flow to the appropriate ward [32]. A stroke protocol was the only enabler that was reported in relation to facilitating appropriate triage [31], and similarly, efficient processes was one of the three enabling factors reported to assist in the implementation of a swallow screen tool [24].

#### Social influences

Social influences were reported in relation to the swallow and tPA related behaviours. A lack of support was reported as a barrier in two studies in relation to the tPA elements [28, 29]. Enablers associated with social influences included sharing of experiences amongst staff [28], the endorsement of the use of tPA [25], and advice from a senior colleague [25]. Social support provided from administrators and the wider multidisciplinary team was perceived by nurses as factors which would assist the uptake of swallow screening practices in ED [24].

#### Behaviour regulation

Data were only reported for the tPA-related behaviours. One reported enabler relevant to this domain was initiation of an assurance mechanism with continuous feedback on success or otherwise of implementation [28].

No authors attempted to interpret the data using an existing behaviour change theory, framework, or model. A purposefully developed taxonomy was used in one study to classify barriers [27], whilst in one other study enablers were classified into behaviour change domains; however, the framework, model or theory that underpinned these domains was not disclosed [25]. A summary of the TDF domains as represented by the barriers and enablers reported in the studies is shown in Table 6.

**Table 6:**
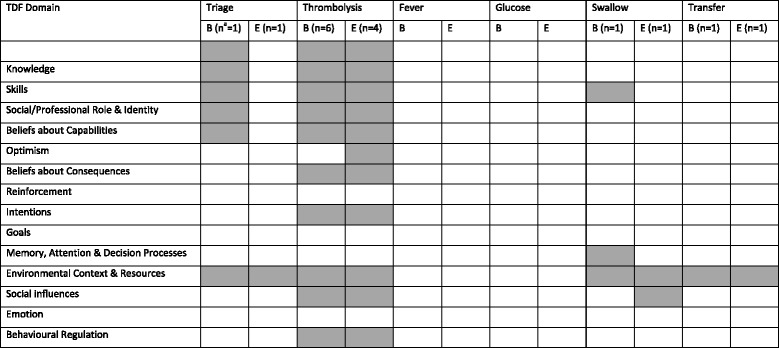
Barriers and enablers classified by TDF domain by target clinical behaviour

^a^Number of studies. *B* Barriers, *E* Enablers. Note: no studies were identified which addressed the care elements relating to temperature and blood glucose level monitoring and management

### The allocation of BCT labels to reported enablers

The total number of BCTs assigned was 18. The BCTs most frequently assigned to the reported enablers were ‘focus on past success’ (*n* = 8) and ‘information about health consequences’ (*n* = 5). There were three occasions where more than one technique was considered to align with a reported enabler. No technique was aligned with the reported enabler “positive staff attitudes, within and outside the stroke unit” as the underlying meaning was not clear and no other contextual data was provided in the study. The aligning behaviour change techniques for each reported enabler are provided in Table 5.

## Discussion

This study produced new knowledge on the barriers and enablers for the delivery of key evidence-based protocols in an emergency setting and interpreted within a relevant theoretical framework. Barriers and/or enablers were identified for triage, thrombolysis, swallow assessment and patient transfer related clinical behaviours. Barriers relating the *environmental context and resources* domain such as a lack of time, stressful working conditions and ED delays were common to all four behaviours. Barriers relating to the *skills* domain such as inconsistent administration of swallow screen tools, and to the *memory*, *attention*, *and decision processes* domain such as difficulty recalling screening items during administration were relevant to only one clinical behaviour. There were no studies which identified barriers or enablers with regards to two of the six target behaviours: monitoring and management of (1) temperature and (2) blood glucose levels. There were no barriers or enablers derived from the systematic review findings that could not be accounted for by one of the TDF domains, indicating that this framework is highly relevant to behaviour change within this clinical context.

The use of the TDF has allowed the comprehensive identification of barriers and enablers for areas such as these where existing evidence is lacking. This process has identified several domains of the TDF where no primary barriers or enablers were identified in the included studies: *reinforcement*, *goals*, and *emotion*. As significant emotional issues such as excessive fear of harm and complications have been identified in other literature [37, 38] regarding thrombolysis in stroke, this emphasises that there remain gaps the published literature where barriers and enablers have not been reported. Evidence suggests that individuals find it more difficult to verbalise their affective attitudes (i.e., emotions) [39]. The TDF has been found to elicit emotion related barriers to behaviour change more effectively than a-theoretical approaches to identifying barriers [7].

Interestingly, only one included study subsequently used the data from a barrier and enabler assessment to develop an implementation intervention (a dysphagia screening bundle) [24]. The evaluation revealed that swallowing screening practices significantly improved after the implementation of this bundle; however, the author did not state whether this could be attributed to the information yielded by the barrier assessment. Indeed, the findings from this systematic review have since been used to inform the development of an implementation intervention for an ongoing trial (the T^3^ trial) to target these clinical behaviours in an ED setting. The T^3^ trial investigators aim to evaluate the supported implementation of key best practice acute stroke care clinical elements relating to the appropriate triage, treatment with tPA, management and monitoring of blood glucose levels, temperature and of swallowing difficulties, and transfer from ED to the ASU.

Although barriers and enablers are often hypothesised to be determinants of behaviour, their actual influence on performing a certain practice has been questioned as other contextual factors may play more significant roles [40]. The large number of barriers and enablers within the *environmental context and resources* domain highlight the importance of health system environments. A broader health systems approach has been used to investigate the association between health system factors such as urban location and tPA treatment rates [41]. Paul et al. concluded that to improve tPA treatment rates, specific health system factors need to be targeted.

### Limitations

There were few studies that met the inclusion criteria for this review included in this review, most likely due to the limited published literature in this field. Due to the limitation of subject headings for the terms “barriers” and “enablers,” electronic indexing synonyms were used for key words to ensure the search was sensitive; however, this may have compromised the precision of the search and as for other reviews of barriers and enablers in healthcare there is potential for studies not to be identified [42]. The quality of the included studies was comprised by small sample sizes for the quantitative studies and the limited reporting and/or lack of robust sampling techniques. The different approaches used to analyse the qualitative and quantitative data within the included studies posed challenges for data extraction. For example, some of the studies referred to stroke care in general, making it difficult to ascertain which barriers related to each of the target clinical behaviours. The use of the TDF for secondary analysis required some subjective interpretation due to the lack of contextual detail reported for some of the barriers and enablers. This limitation has been reported by other authors [21]. No included study used a behaviour change theory, standard taxonomy, or framework to interpret findings.

Researchers have reported that barriers can be represented by more than one domain and have also acknowledged the complex relationship between domains [21]. In this study this was evident when attempting to interpret and map the barriers to the framework with often limited contextual information. For example, “inaccurate interpretation of screening items” in performing a swallow screen was mapped to *skills* as the primary TDF domain but may also be related to the TDF domain *memory*, *attention*, *decision processes*. In this study, we agreed and reported the primary domain only. Further, research is required to guide how best to select a theoretical domain in these instances, e.g., what process should be taken to identify which of two (or more) domains primarily represents a specific barrier.

There were instances whereby the BCT used in the enabling strategy reported in the included studies did not align with the BCTs that the Cane et al. matrix recommends for use to target domains. For example, the BCTs used in the enabling strategies (habit formation; feedback on outcome of behaviour; feedback on behaviour) for the behavioural regulation domain do not align with the BCT recommended by the Cane et al. matrix (self-monitoring of behaviour). This indicates that Cane et al. matrix is a useful, but not comprehensive tool to identify appropriate enabling strategies. It is likely that the discrepancies are due to individual contextual factors, not accounted for by the matrix; highlighting the ongoing importance of local barrier and enabler assessments for any implementation strategy.

### Strengths

Both qualitative and quantitative primary studies were focused on capturing barriers and enablers as reported by healthcare professionals. This provides novel data in comparison to existing reviews in this area which often relied on observational or registry data to characterise barriers, i.e., investigate causes or consequences of pre-determined factors such as in-hospital delays. The review processes used were robust and transparent adhering to the Preferred Reporting Items for Systematic Reviews and Meta-Analyses (PRISMA) standard. The search strategy underwent extensive review and iteration to ensure efficiency and accuracy in the conduct and output of the search. The data collection process was conducted in duplicate and decisions were cross-checked with another researcher.

The use of the TDF in systematic reviews is an emerging methodology and has only been applied in a few recent studies. The assignment of BCTs to barriers was not conducted as part of this review as the information was not available in the individual studies. However, one potential application of the barrier data is to select; using a panel of experts for example, the most appropriate behaviour change techniques to address the barriers to further inform the T^3^ trial implementation intervention development. For example, one of the BCTs recommended by the mapping conducted by Cane et al. [18] to overcome barriers classified within the *skills* domain is behavioural practice/rehearsal [35]. The enabler data from this review could then be applied to inform how this technique can be feasibly employed in clinical practice. For example, the enabler “giving physicians the opportunity to treat stroke cases of varying complexity” could be viewed as a deliverable form of this technique. This approach has been successfully used to develop an implementation intervention to improve the management of traumatic brain injury in the ED [43]. Furthermore, classifying the enablers to the best aligning BCT enhances the reportability and transferability of findings in this process.

### Area of future research

The authors of one survey presented and analysed the data by decision makers and non-decision makers and revealed significant difference between the two groups [25]. Therefore, it may be important to further identify and explore the differences and similarities in perceptions between different disciplines, especially when implementing a multidisciplinary intervention. It would be worthy to further investigate whether the barriers identified by the review could be feasibly addressed, possibly by devising a panel of experts to make this judgment. If the barrier is deemed non-modifiable, then it is not likely to be a feasible target for an implementation intervention. The monitoring of barrier status during the implementation of an intervention would provide evidence for the types of barriers that can easily be overcome, the type of barriers which may be intractable and the effectiveness of particular BCT directed at barriers/enablers within various theoretical domains. Currently, mapping of BCTs to the TDF domains is largely based on expert opinion and a more robust higher level of evidence is required. There also needs to be more illustrations of how barrier and/or enabler data has been used to develop implementation interventions. Consistent use of a theoretical framework will assist with progressing this work by enabling meaningful compilation of evidence from a variety of sources. In addition, none of the studies reported monitoring the status of barriers over time or conducted a pre-and post implementation barrier assessment. Such design would add significant weight to the evidence regarding the value of barrier and enabler assessment, and subsequent use to target interventions.

Gaining patient’s and family member’s perceptions of stroke care in the ED were not considered by any of the studies and in the context of shared-decision making is an area that warrants further research. Patient and family preferences for and against practices such as tPA [44] and “nil by mouth” [45] has the potential to impact on the implementation of certain practices.

## Conclusion

Barriers and/or enablers have been identified for the majority of the target clinical behaviours which could be used to inform barrier and enabler assessments in similar acute settings. Due to the likely gaps in the evidence base, barrier, and enabler data for some of the clinical behaviours and within some of the theoretical domains, could not be identified. The novel assignment of BCT labels to reported enablers will allow researchers and clinicians to use and potentially modify these techniques to deliver these important clinical behaviours in routine practice. When considering the findings from reviews of barriers and enablers, it is recommended that a reporting framework as illustrated in this paper should be adopted. This would facilitate the comparison, contrasting, and synthesis of barrier and/or enabler data within a consistent context framework and facilitate recognition identification of proven strategies to address such barriers [19].

## Appendix

### Search Strategies

#### OVID Medline

1. exp cerebrovascular disorders/or exp basal ganglia cerebrovascular disease/
2. exp basal ganglia hemorrhage/or exp brain ischemia/
3. exp brain infarction/or exp brain stem infarctions/
4. exp cerebral infarction/or exp infarction, anterior cerebral artery/
5. exp infarction, middle cerebral artery/
6. exp infarction, posterior cerebral artery/
7. exp hypoxia-ischemia, brain/
8. exp “intracranial embolism and thrombosis”/
9. exp intracranial hemorrhages/
10. exp cerebral hemorrhage/
11. exp subarachnoid hemorrhage/or stroke/
12. exp Health Plan Implementation/
13. *“Diffusion of Innovation”/
14. ((implementation or implementing) and (care or healthcare)).ti.
15. ((effect? or effectiveness or chang$ or improv$ or impact) adj3 practice).ti.
16. (Improv$ adj3 (diagnosis or treatment? or prescribing)).ti.
17. Evidence-Based Practice/
18. (((evidence or evidence-based) adj4 intervention) or evidence-driven).ti,ab.
19. ((knowledge adj2 (application or broke$ or creation or diffus$ or disseminat$ or exchang$ or implement$ or management or mobili$ or translat$ or transfer$ or uptake or utili$)) or (evidence$ adj2 (exchang$ or translat$ or transfer$))).ti.
20. “Quality of Health Care”/
21. *Delivery of Health Care/st [Standards]
22. *Treatment Outcome/
23. “Continuity of Patient Care”/
24. (determin$ or facilitate$ or barrier$ or enabler$).ti.
25. (evidence$ adj2 (barrier$ or enabler$ or enabler$ or uptake$ or utilis$ or utiliz$ or diffus$ or disseminat$)).tw.
26. ((information or evidence) adj2 uptake).ti.
27. (research adj2 (barrier$ or enabler$ or enabler$ or uptake$ or utilis$ or utiliz$ or diffus$ or disseminat$)).tw.
28. (information adj2 (barrier$ or enabler$ or enabler$ or uptake$ or utilis$ or utiliz$ or diffus$ or disseminat$)).tw.
29. (data adj2 (barrier$ or enabler$ or enabler$ or uptake$ or utilis$ or utiliz$ or diffus$ or disseminat$)).tw.
30. Health Services Accessibility/
31. *Critical Pathways/og [Organization & Administration]
32. Clinical Protocols/
33. Models, Organizational/
34. exp triage/
35. exp delegation, professional/
36. (stroke adj3 (unit or units or ward or wards or hospital or hospitals or centre$ or team or teams)).tw.
37. (“early transfer” or “early referral”).ti.
38. Decision Making/
39. Early Diagnosis/
40. Emergency Nursing/
41. *Stroke/di [Diagnosis]
42. *Stroke/nu [Nursing]
43. *Stroke/dt [Drug Therapy]
44. Emergency Service, Hospital/og [Organization & Administration]
45. *Time Factors/
46. exp Tissue Plasminogen Activator/
47. exp thrombolytic therapy/
48. exp fibrinolytic agents/or plasmin/or plasminogen/or tissue plasminogen activator/
49. ((clot$ or thrombus) adj5 (lyse or lysis or dissolve$ or dissolution)).ti,ab.
50. (tPA or t-PA or rtPA or thrombolysis or plasminogen or plasmin or alteplase or actilyse).ti,ab.
51. (anistreplase or streptodornase or streptokinase or urokinase or pro?urokinase or rpro?uk or lumbrokinase or duteplase or lanoteplase or pamiteplase or reteplase or saruplase or staphylokinase or streptase or tenecteplase or desmoteplase or retevase).tw.
52. exp fever/
53. (fever or temperature or hyperthermia or pyrexia).tw.
54. exp Blood Glucose/
55. Hyperglycemia/di [Diagnosis]
56. Hyperglycemia/dt [Drug Therapy]
57. *Hyperglycemia/th [Therapy]
58. Hypoglycemic Agents/ae [Adverse Effects]
59. Hypoglycemic Agents/tu [Therapeutic Use]
60. *Monitoring, Physiologic/nu [Nursing]
61. exp insulins/or insulin infusion systems/
62. *Deglutition Disorders/
63. (swallow$ or dysphagia).ti.
64. 13 or 14 or 15 or 16 or 17 or 18 or 19 or 20 or 21 or 22 or 23 or 24 or 25 or 26 or 27 or 28 or 29
65. 1 or 2 or 3 or 4 or 5 or 6 or 7 or 8 or 9 or 10 or 11
66. 12 or 13 or 14 or 15 or 16 or 17 or 18 or 19 or 20 or 21 or 22 or 23 or 24 or 25 or 26 or 27 or 28 or 29
67. 30 or 31 or 32 or 33 or 34 or 35 or 38 or 39 or 40 or 41 or 42 or 43 or 44 or 45 or 46 or 47 or 48 or 49 or 58 or 51 or 52 or 53 or 54 or 55 or 56 or 57 or 58 or 59 or 60 or 61 or 62 or 63
68. 66 and 67 and 68
69. Letter/or Comment/or Editorial/
70. 69 not 70

#### OVID EMBASE Search Strategy

1. exp clinical pathway/
2. ((implementation or implementing) and (care or healthcare)).ti.
3. ((effect? or effectiveness or chang$ or improv$ or impact) adj3 (practice or organisation$)).ti.
4. (Improv$ adj3 (diagnosis or treatment?)).ti.
5. *evidence based practice/
6. (((evidence or evidence-based) adj4 intervention) or evidence-driven).ti.
7. ((knowledge adj2 (application or broke$ or creation or diffus$ or disseminat$ or exchang$ or implement$ or management or mobili$ or translat$ or transfer$ or uptake or utili$)) or (evidence$ adj2 (exchang$ or translat$ or transfer$))).ti.
8. *health care delivery/
9. *Treatment Outcome/
10. *“Continuity of Patient Care”/
11. (evidence$ adj2 (barrier$ or enabler$ or enabler$ or uptake$ or utilis$ or utiliz$ or diffus$ or disseminat$)).tw.
12. ((information or evidence or implement$) adj2 (uptake or determin$ or enabler$ or barrier$ or enabler$)).ti,ab.
13. (research adj2 (barrier$ or enabler$ or enabler$ or uptake$ or utilis$ or utiliz$ or diffus$ or disseminat$)).tw.
14. (information adj2 (barrier$ or enabler$ or enabler$ or uptake$ or utilis$ or utiliz$ or diffus$ or disseminat$)).tw.
15. (data adj2 (barrier$ or enabler$ or enabler$ or uptake$ or utilis$ or utiliz$ or diffus$ or disseminat$)).tw.
16. exp triage/
17. exp delegation, professional/
18. (stroke adj3 (unit or units or ward or wards or hospital or hospitals or centre$ or team or teams)).tw.
19. (“early transfer” or “early referral”).ti.
20. Decision Making/
21. Early Diagnosis/
22. Emergency Nursing/
23. stroke/di, dt, th [Diagnosis, Drug Therapy, Therapy]
24. emergency health service/
25. *time/
26. Tissue Plasminogen Activator/
27. exp thrombolytic therapy/
28. exp fibrinolytic agents/or plasmin/or plasminogen/or tissue plasminogen activator/
29. ((clot$ or thrombus) adj5 (lyse or lysis or dissolve$ or dissolution)).tw.
30. (tPA or t-PA or rtPA or thrombolysis or plasminogen or plasmin or alteplase or actilyse).tw.
31. (anistreplase or streptodornase or streptokinase or urokinase or pro?urokinase or rpro?uk or lumbrokinase or duteplase or lanoteplase or pamiteplase or reteplase or saruplase or staphylokinase or streptase or tenecteplase or desmoteplase or retevase).tw.
32. fever/
33. (fever or temperature or hyperthermia or pyrexia).ti.
34. exp Blood Glucose/
35. hyperglycemia/di, dt, rh, si [Diagnosis, Drug Therapy, Rehabilitation, Side Effect]
36. antidiabetic agent/ae, th [Adverse Drug Reaction, Therapy]
37. insulin derivative/
38. dysphagia/
39. (swallow$ or dysphagia).ti.
40. exp cerebrovascular disorders/
41. ((cerebral* or cerebellar or brainstem or vertebrobasilar or stroke) adj5 (infarct* or ischaemi* or ischemi* or thrombo* or emboli* or apoplexy)).tw.
42. exp hemiplegia/
43. (hemipleg* or hemipar* or paresis or paretic).tw.
44. (stroke or poststroke or post-stroke or cva* or cerebral vascular accident* or cerebrovascular accident*).tw.
45. ((cerebral or brain or subarachnoid) adj5 (haemorrhage* or hemorrhage* or haematoma* or hematoma* or bleed*)).tw.
46. 1 or 2 or 3 or 4 or 5 or 6 or 7 or 8 or 9 or 10 or 11 or 12 or 13 or 14 or 15
47. 16 or 17 or 20 or 21 or 22 or 23 or 24 or 25 or 26 or 27 or 28 or 29 or 30 or 31 or 32 or 33 or 34 or 35 or 36 or 37 or 38 or 39
48. 40 or 41 or 42 or 43 or 44 or 45
49. 46 and 47 and 48
50. Letter/or Editorial/
51. 49 not 50

#### CINHAL

1. (MH “Cerebrovascular Disorders”) OR (MH “Basal Ganglia Cerebrovascular Disease+”) OR (MH “Carotid Artery Diseases+”) OR (MH “Cerebral Ischemia+”) OR (MH “Cerebral Vasospasm”) OR (MH “Intracranial Arterial Diseases+”) OR (MH “Intracranial Embolism and Thrombosis”) OR (MH “Intracranial Hemorrhage+”) OR (MH “Stroke”) OR (MH “Vertebral Artery Dissections”)
2. (MH “Stroke Patients”)
3. TI (stroke or cerebrovasc* or brain vasc* or cerebral vasc* or cva* or apoplex*) or AB (stroke or cerebrovasc* or brain vasc* or cerebral vasc* or cva* or apoplex*)
4. TI (brain* or cerebr* or cerebell* or vertebrobasilar or hemispher* or intracran* or intracerebral or infratentorial or supratentorial or MCA or anterior circulation or posterior circulation or basal ganglia) or AB (brain* or cerebr* or cerebell* or vertebrobasilar or hemispher* or intracran* or intracerebral or infratentorial or supratentorial or MCA or anterior circulation or posterior circulation or basal ganglia)
5. TI (ischemi* or ischaemi* or infarct* or thrombo* or emboli*) or AB (ischemi* or ischaemi* or infarct* or thrombo* or emboli*)
6. TI (brain brain* or cerebr* or cerebell* or intracerebral or intracran* or parenchymal or intraventricular or infratentorial or supratentorial or basal gangli*) or AB (brain* or cerebr* or cerebell* or intracerebral or intracran* or parenchymal or intraventricular or infratentorial or supratentorial or basal gangli*)
7. TI (haemorrhage* or hemorrhage* or haematoma* or hematoma* or bleed*) or AB (haemorrhage* or hemorrhage* or haematoma* or hematoma* or bleed*)
8. MJ implementing
9. TI (uptake or determine$ or enabler$ or enable$ or barrier$)
10. S8 OR S9
11. S1 OR S2 OR S3
12. S4 AND S5
13. S6 AND S7
14. S11 OR S12 OR S13
15. S15 AND S10

#### Web of Science

1. TS = (stroke or cva or “cerebral vascular” or cerebrovascular)
2. TI = (information OR evidence OR implement*)
3. TI = (uptake or determin$ or enabler$ or enable$ or barrier$)
4. #2 and #3
5. #1 AND #4

## Abbreviations

ASU: Acute stroke unit
ED: Emergency Departments
T^3^: Triage, Treatment and transfer
TDF: Theoretical domains framework
tPA: Thrombolysis
